# Improvement of quality of life in methadone treatment patients in northern Taiwan: a follow-up study

**DOI:** 10.1186/1471-244X-13-190

**Published:** 2013-07-16

**Authors:** Ying-Chun Chou, Shu-Fang Shih, Wei-Der Tsai, Chiang-shan R Li, Ke Xu, Tony Szu-Hsien Lee

**Affiliations:** 1 Department of Health Promotion and Health Education, National Taiwan Normal University, No. 162, He-Ping East Road, Section 1, Taipei 10610, Taiwan; 2 Graduate Institute of Industrial Economics, National Central University, No.300, Jhongda Road, Jhongli City, Taoyuan County 32001, Taiwan; 3 Department of Psychiatry, Yale University School of Medicine, 34 Park Street, New Haven 06519, USA

**Keywords:** Effectiveness, Heroin users, Methadone maintenance treatment, Quality of life

## Abstract

**Background:**

This study examined long-term improvement of quality of life amongst heroin users enrolled in methadone maintenance treatment (MMT).

**Methods:**

The sample contained 553 heroin-dependent individuals from 4 hospitals in northern Taiwan who enrolled in MMT for an average of 184 days. Each patient signed a consent form and was assessed prospectively 3 times semi-annually. Quality of life was measured using the WHOQOL-BREF questionnaire, 26 items of which were scored by the participants. The WHOQOL-BREF consists of four domains: physical, psychological, social, and environmental. 285 and 155 participants completed 6-month and 12-month follow-ups respectively.

**Results:**

After controlling for demographic and clinical characteristics, there were statistically significant improvements in the psychological and environmental domains between baseline and 6 months. Significant improvements were found in psychological and social domains between baseline and 12 months.

**Conclusions:**

It is concluded that methadone maintenance treatment improves heroin users’ long-term quality of life in the psychological and social relationship domains.

## Background

Illicit drug use is a complicated problem that not only impacts individuals’ physical and psychological health but also derogates public security and society’s productivity because of morbidity and mortality [1]. It is estimated that about 230 million people, or 5% of the world’s adult population, used an illicit drug at least once in 2010 [2]. The report also estimates that heroin, cocaine, and other drugs kill around 0.2 million people each year, shattering families and bringing misery to thousands of other people.

Heroin users are at high risk of morbidity and mortality. They may struggle with dependence for many years, suffering poor interpersonal relationships and poor health [3,4], particularly due to overdosing [5] and human immunodeficiency virus (HIV) and hepatitis C virus (HCV) infections [6,7]. A national study conducted in Taiwan on 22,224 male and 4,444 female drug offenders released from prisons without medical treatment shows that the standardized mortality ratio was seven to one, much higher than for the general population [8].

Follow-up studies have found that this risk continues for many years after diagnosis of heroin dependence [9], indicating that the dependence can be considered chronic. In fact, heroin addiction is currently defined as a chronic, relapsing disorder [10,11]. Methadone, a complete opioid agonist, has been approved as a treatment for heroin addiction, especially with higher doses [12,13] because it can help patients reduce their frequency of heroin use, alleviate withdrawal symptoms, and consequently improve overall health and social relationships [14-16]. Thus, heroin users taking methadone should be able to improve cognitive functions [17] and return to normal daily activities. This means that we would expect quality of life amongst heroin users to improve during treatment [18]. However, according to the National Epidemiologic Survey on Alcohol and Related Conditions, fewer than 40% of those diagnosed with drug dependence in the United States have ever received any kind of intervention or treatment. In Taiwan, a methadone maintenance treatment (MMT) program was initiated to prevent HIV, and the program had expanded to the entire island by the end of 2006. Currently, about 12,000 out of the 60,000 heroin users in Taiwan participate in an MMT program everyday [19].

Although opioid treatment programs are effective in reducing the amount and frequency of heroin use and its consequences as describe above, prospective research on possible improvement in quality of life from the patient’s perspective has been limited. Quality of life was defined by the WHO as “an individual’s perception of their position in life, in the context of the culture and value systems in which they live and in relation to their goals, expectations, standards and concerns”. Quality of life has been an important indicator of efficacy in the treatment of various chronic diseases such as diabetes and hypertension, creating a transition from cure to care. In their report of a qualitative interview study, Laudet, Becker, and White [20] noted that the heroin users they studied had been suffering considerable negative consequences from their habit for a very long time. It is common for heroin users to prefer a return to a normal life, or just a better life, over the chaotic, abnormal life they had been undergoing. Although published studies in the addiction field on quality of life remain sparse, the few that have appeared consistently show that MMT can improve heroin users’ quality of life within a short period of time [21-23]. For example, Maremmani, Oani, Pacini and Perugi [21] studied 213 patients (106 on buprenorphine and 107 on methadone) between the 3rd and 12th months of treatment for both medications and found significant improvements in opioid use, psychiatric status, and quality of life. On the other hand, the authors of a recent review of 38 articles concluded that the long-term effects of treatment on the quality of life of heroin using patients remain unclear [24].

Further, MMT is not free to patients. A recent study on 2,728 MMT patients in China [25] indicated that perception of suitable MMT cost is a significant predictor of retention. Moreover, in another study [13] that used a signal detection analysis on 258 methadone outpatients to identify predisposing factors that predict treatment retention, the results showed that satisfaction of treatment is a significant predictor. Additionally, Taiwanese heroin users have to pay for the medical expenses, which can be directly related to their satisfaction, participation and retention in MMT. By comparing the changes of quality of life at baseline, 6-month and 12-month follow-up survey, we could evaluate the effectiveness of MMT on quality of life and the monetary costs for per unit change of quality of life gained. Even though MMT programs are relatively new in Asia except Hong Kong, we hypothesized that MMT patients can improve long-term subjective quality of life of Taiwanese heroin users 6 and 12 months after enrollment in this study. Furthermore, the ratio of costs over per unit increment of quality of life may provide a useful index to further evaluate patient’s perception of suitable costs and treatment satisfaction and form a basis of policy decision making.

## Methods

### Study design

We used a 12-month prospective multisite study to examine the improvement of quality of life from the patient’s perspective. The study protocol was reviewed and approved by the institutional review board (IRB) of Taipei Medical University (approval number: P960205) and by Taipei City Hospital (approval number: TCHIRB-970404-E). This was an open, nonrandomized, and observational study.

### Participants

Participants were recruited from four outpatient MMT clinics in northern Taiwan during the years 2008 and 2009. Each patient at admission self-reported his/her history of drug use, and underwent morphine, HIV and HCV tests. Patients paid the associated medical costs of each MMT service utilized during his/her duration of treatment, including registration, diagnosis, various tests, prescription, medication as well as health education, with the exception of those who were HIV positive. Per Taiwan’s policy, HIV-positive patients were reimbursed for all medical costs from Taiwan Department of Health. Counseling and/or psychotherapy may be offered only on demand. The inclusion criteria in this study were age above 20 years, heroin dependence, and literacy. After signing a consent form, each patient was interviewed face-to-face by a trained research assistant at three times: baseline, 6 months into the study, and 12 months into the study. Each patient was reimbursed approximately 3 US dollars for each interview.

### Quality of life

Based on definition of quality of life, we used the World Health Organization Quality of Life Assessment-Brief Version (WHOQOL-BREF), which was developed to measure overall quality of life and general health status [26,27]. The WHOQOL-BREF measures four health domains: physical, psychological, social, and environmental. The WHOQOL-BREF (Taiwanese version) has shown excellent internal consistency (Cronbach’s alpha = .97) and good test-retest reliability over 2 weeks for the four domains (.68 to .85) [27]. The four-domain structure was validated by exploratory and confirmatory factor analyses, with factor loadings > 0.40 for the retained items and CFI > 0.90 [27].

The WHOQOL-BREF questionnaire uses a five-point Likert scale (1–5). Four types of scale descriptors (capacity, frequency, intensity, and evaluation) were selected for this study. Participants responded to questions according to their experience in the previous two weeks. Reverse items (items 3, 4 and 26) were scored reversely. Domain scores for the WHOQOL-BREF are calculated by taking the mean of all items included in each domain and multiplying by a factor of four. The original domain scores were transformed to a scale of 0–100 according to the equation in the published guidelines [26,27]. A high score represents good quality of life. An increase in QoL scores after 6- or 12-month treatment would suggest the effectiveness of MMT. Furthermore, given that each service of MMT has a fixed unit price regulated by Taiwan Department of Health, the total medical costs of MMT were computed by summing up the unit prices of all the services utilized by a patient during his/her 12-month retention (see Table 1). Medical costs borne by patients for one QoL score increment can be estimated by dividing the costs by the changes of QoL scores.

**Table 1:** Costs associated with medical services and examinations in MMTP in 2009 placarded by Taiwan Department of Health

**Medical items**	**Unit cost (US dollars)**	**12-month cost (US dollars)**
First-visit diagnostic fees	153.3	153.3
Registration fees	5	75
Prescription fees	10	120
Examination fees		
HIV test semiannually	13.3	26.7
Urine test (Morphine)	10	20
Urine test (Amphetamine)	10	20
HBsAg test	8.3	16.7
Anti-HBs test	10	20
Anti-HCV test	11.7	23.3
TPHA test (Treponemapallidumhemagglutination)	13.3	26.7
SGOT blood test	1.7	3.3
SGPT blood test	1.7	3.3
r-GT test	2.3	4.7
Chest X-ray	10	20
EKG (Electrocardiograph)	0	0
Supportive group education	10	120
Psychotherapy (Family therapy, group therapy, or other psychotherapies)	106.7	106.7
Healthcare service fees (including health education and counseling)	0	0
Case management service fees	166.7	166.7
Medication fee (methadone per day)	0.7	243.3
Total		1179.7

*Note*. Medical payments were collected according to the regulations issued by the Taiwan Department of Health in 2009 governing payments for prevention, testing, and treatment of HIV.

### Statistical analysis

Descriptive statistics were used to identify the characteristics of the entire sample and the two groups of patients who stayed in or dropped out of MMT during the 6-month study period. Chi-square and *t*-test analyses were performed on categorical and continuous variables respectively to determine if there were significant differences between the two groups. Quality of life scores, age, age at first heroin use, education, methadone dose, and duration in treatment in this study met the normality assumption. To better estimate the associations among the four domains of the quality of life between assessment waves, repeated measures analyses of variance (ANOVA) with a 0.05 significance level for longitudinal evaluations were performed to examine changes on the four domains simultaneously while controlling for age, education, gender, age at first heroin use, methadone dose and days stayed in MMT.

## Results

A total of 599 patients who enrolled in an MMT program for an average of 184 days signed the consent form and 46 did not go through the baseline assessment. Of the 553 of those who completed baseline assessment, 285 and 155 remained in the study each for 6 and 12 months.

Table 2 summarizes the background information and compares participants who retained in the study for the 6-month follow-up and those who dropped out in 6 months after baseline. No difference from chi-square and *t*-test analysis was found for gender, age, marital status, education, age at first heroin use, HIV, HCV, whether morphine was tested positive at baseline, methadone dose at treatment admission, employment, and number of days enrolled in the MMT before enrollment in this study.

**Table 2:** Comparison of study participants who retained in MMT and those who dropped out of MMT at 6-month follow-up

	**Retained in MMT at 6-month follow-up (*n*** **= 285)**		**Dropped out of MMT at 6-month follow-up (*n*** **= 314)**		***χ***^**2**^**/*t***	***p***
	***n*(%)**	***M*( *SD *)**	***n*(%)**	***M*( *SD *)**		
Gender					0.49	0.48
Male	245(86.0)		276(87.9)			
Female	40(14.0)		3(12.1)			
Age		41.02(9.32)		40.16(9.09)	1.15	0.25
Marital Status	(*n* = 284)		(*n* = 263)		4.12	0.25
Married	85(29.9)		61(23.2)			
Single	141(49.7)		149(56.6)			
Divorced/Widowed/Other	58(20.4)		53(20.2)			
Education (years)		9.22(2.26)	(*n* = 309)	9.1(2.17)	0.29	0.77
Age at first-time heroin intake	(*n* = 283)	27.74(7.36)	(*n* = 218)	26.65(7.34)	1.65	0.10
HIV status	(*n* = 275)		(*n* = 301)		0.01	0.93
Positive	34(12.4)		38(12.6)			
Negative	241(87.6)		263(87.4)			
HCV Status	(*n* = 264)		(*n* = 287)		1.11	0.58
Positive	248(93.9)		264(92.0)			
Negative	16(6.1)		23(8.0)			
Morphine status at baseline	(*n* = 247)		(*n* = 266)		2.55	0.28
Positive	69(27.9)		90(33.8)			
Negative	178(72.1)		176(66.2)			
Methadone dose at treatment intake (mg/day) (SD)	(*n* = 283)	39.19 (20.86)	(*n* = 313)	38.58 (19.90)	0.37	0.72
Days enrolled in MMT before this study (SD)	(*n* = 216)	194.44 (157.86)	(*n* = 282)	175.77 (145.10)	1.37	0.17
Employed	(*n* = 279)		(*n* = 292)		0.44	0.51
Yes	187 (67.0)		188(64.4)			
No	92(33.0)		104(35.6)			

*Note*. Variables with missing values were deleted from the analyses. Chi-square and *t*-test analyses were performed on categorical and continuous variables respectively to determine if there were differences between dropouts and those who stayed.

Table 3 lists the WHOQOL-BREF scores at baseline and at 6-month for participants who completed the quality-of-life questionnaire at both times. At baseline, the highest mean score was in the physical domain (58.53; *SD* = 15.51) and the lowest mean score was in the psychological domain (49.89; *SD* = 16.64). Likewise, at 6 months, the highest mean score was in the physical domain (60.13; *SD* = 14.68) and the lowest was in the psychological domain (53.19; *SD* = 17.15).

**Table 3:** **Quality-of-life (QoL) scores and cost per QoL point in the four domains at the 6-month assessment (*n*** **= 285)**

	**Baseline score**	**6-month score**	**QoL gained**	**Cost per QoL point**
	***M *( *SD *)**	***M *( *SD *)**		
Physical	58.53(15.51)	60.13(14.68)	1.6	368.7
Psychological	49.89(16.64)	53.19(17.15)	3.3^**^	178.7
Social Relations	54.71(18.13)	55.77(17.05)	1.06	556.5
Environmental	52.92(16.97)	55.42(16.20)	2.5^**^	235.9

*Note*. Repeated measures analyses of variance of baseline and 6-month follow-up were performed to control for age, education, gender, age at first heroin use, methadone dose and days in MMT before study. Cost per QoL point = Total MMT cost/Incremental QoL gain. MMT cost was $1179.70 in 2009. For the 6-month calculation, $589.85 (1179.7/2) was used.

^*^p < .05.^**^p < .01.

To assess changes in quality of life, the scores at baseline were subtracted from the scores at 6 months. Results from repeated measures analyses of variance of baseline and 6-month follow-up showed that age, education, gender, age at first heroin use, methadone dose and days in MMT before study were not significantly associated with changes of quality of life. Table 3 shows that the greatest mean gain at 6-month follow-up was in the psychological domain (3.3) and the least in the social domain (1.06). Significant improvements were found in the psychological domain, *F*(1, 284) = 3.66, *p* < .01, and the environmental domain, *F*(1, 284) = 2.68, *p* < .01. When the gained quality of life scores were divided by the total MMT cost (approximately 1179.7 US dollars) that a patient had to pay in the year of 2009 (see Table 1), the results indicated that at 6 months the cost was lowest for improvement in the psychological domain ($178.70/per quality-of-life (QoL) unit).

Results from repeated measures analyses of variance of baseline, 6-month and 12-month follow-ups also showed that age, education, gender, age at first heroin use, methadone dose and days in MMT before study were also not significantly associated with changes of quality of life. Table 3 presents the changes in quality of life and the cost in dollars of each point increase in the QoL mean scores between baseline, 6 months, and 12 months. For patients who completed the questionnaire at all three times (*n* = 155), the greatest mean increases in QoL scores were in the psychological domain: baseline to 6 months (2.84) and baseline to 12 months (3.95). In the social domain, the corresponding mean increases were 1.27 from baseline to 6 months and 3.02 from baseline to 12-months. The lowest increase (1.58) was in the physical domain from baseline to 12 months.

Table 4 also shows statistically significant improvements from baseline to 6 months in the psychological domain, *F*(1, 154) = 2.35, *p* < .01, and in the environmental domain, *F*(1, 154) = 2.00, *p* < .01. From baseline to 12 months, there were significant improvements in the psychological domain, *F*(1, 154) = 3.05, *p* < .01,and in the social domain, *F*(1, 154) = 1.95, *p* < .01).

**Table 4:** **Quality-of-life (QoL) scores and cost per QoL point in the four domains at the 6- and 12-month assessments (*n*** **= 155)**

				**Incremental QoL gain**		**Cost per QoL point**	
	**Baseline score**	**6-month**	**12-month**	**6-month**	**12-month**	**6-month**	**12-month**
	***M*( *SD *)**	***M*( *SD *)**	***M*( *SD *)**				
Physical	56.80 (15.14)	58.59 (14.48)	58.38 (14.52)	1.79	1.58	329.5	746.6
Psychological	50.06 (16.23)	52.90 (16.48)	54.01 (17.37)	2.84^*^	3.95^**^	207.7	298.7
Social Relations	53.49 (18.80)	54.76 (17.23)	56.51 (17.85)	1.27	3.02^*^	464.4	390.6
Environmental	52.34 (17.04)	54.79 (16.59)	54.58 (16.80)	2.45^*^	2.24	240.8	526.7

*Note*. Repeated measures analyses of variance of baseline, 6-month and 12-month follow-ups were performed to control for age, education, gender, age at first heroin use, methadone dose and days in MMT before study. Cost per QoL point = Total MMT cost/Incremental QoL gain. MMT cost was $1179.70 in 2009. For the 6-month calculation, $589.85 (1179.7/2) was used.

^*^p < .05. ^**^p < .01.

The gains in quality of life and cost per QoL point gained are also presented in Table 4. The results show that participants had to pay an average of $746.60, $298.70, $390.60, and $526.70 per year in order to gain one QoL point in their physical, psychological, social, and physical domain scores, respectively; note that the least costly was the psychological domain and the most costly was the physical domain.

## Discussion and conclusions

This study examined long-term self-reported improvement in the quality of life of heroin patients in a MMT in Taiwan. Employing a longitudinal design, we found that the MMT improved patients’ quality of life in psychological, social relations, and environmental but not physical domain, as recorded at 6 and 12 months. Specifically, patients who stayed in the program for 6 months (*n* = 285) showed statistically significant improvement in their quality of life in the psychological and environmental domains. Similar positive effects were found in patients who stayed in the program for 12 months (*n* = 155), indicating that these patients showed significant improvement in their psychological health and social relationships.

Overall, our results show that the efficacy of MMT may exist in improving long-term quality of life varied in magnitude and as a function of length of time in the program. Compared with previous studies [21,28], our results show that the gains in mean quality-of-life scores were relatively small at both 6 and 12months. The differences between our study and others in terms of MMT efficacy may be attributed to methadone dose and different sample selection criteria. First, in our study, the average methadone dose was below 40mg/day which is much lower than the recommended dose of at least 59mg/day in previous studies [13]. Second, our sample of patients consisted of drug users who at baseline had already been participating in their MMT, whereas in the other studies the baseline assessment was the patients’ first exposure to MMT. De Maeyer et al. [24] noted that the heroin-dependent persons they studied often found themselves in a crisis situation at the start of treatment and entered treatment in very poor condition, resulting in very poor quality of life at admission. Because it is likely that the marginal effects of MMT on patients’ quality of life decrease the longer they participate in an MMT, it would not be surprising if patients who had never been involved in an MMT program before respond better to MMT than those who had experienced it before. Therefore, the beneficial effects of MMT on quality of life in studies such as those of Xiao [18], Baharom, et al. [28], Maremmani et al. [21], Padaiga et al. [22], Ponizovsky and Grinshpoon [23], and Wang et al. [29] are likely to have covered the time period from patient’s first admission to follow-up treatment. However, findings on the persistence of MMT effects on quality of life have been mixed. Some studies found that patients in treatment for 6 months or longer had a better quality of life than at admission [21-23], whereas Wang et al. [29] and Xiao [18] found that beneficial effects were observed only during the first to third month of treatment. Our results may imply that MMTs improve patients’ quality of life, but the long-term effects might not be as significant as the short-term effects identified by other studies.

In our study, the most significant progress on quality of life was in the mental health area, with an increase of 3.3 in mean scores after 6 months of treatment and 2.84 after 12, in accord with previous studies [28,30]. For instance, Ha [30] found that MMT had a positive impact on the quality of life of drug users in Vietnam after 6 months of treatment; the mean was highest for psychological health (13.28) and lowest for social relationships (3.94). Baharom et al. [28] found Malaysian patients’ quality of life increased significantly in all four domains, with the mean score increases ranging from 7.32 in social relationships to 15.54 in psychological health following 6 months of therapy.

By comparing the beneficial effects of MMT 6 months and 12months after baseline measures, we found that the improvement in social relationships was significant only at 12 months. Padaiga et al. [22] found that the average quality of life of 102 Lithuanian opiate-dependent individuals significantly improved in all the domains except social relationships after 6 months of MMT. One explanation for the lack of effect for social relationships is that it takes time for heroin users to regain trust from non-drug-using families and relatives. MMT not only helps in alleviating the physical harm associated with heroin addiction, but also improves psychological and environmental domains of their lives, as we found in our study at 6 months. With improvements in psychological and environmental well-being, patients would be more willing and able to re-establish social relationships.

On the other hand, we also found that the physical-domain scores of patients did not improve significantly from baseline to 6 months or from 6 to 12 months, although the direction was positive. A possible reason for this failure is that MMT may reduce heroin users’ cravings for drugs and withdrawal symptoms in as short as one month, causing them to feel improvement in their physical status; however, this sense of improvement diminishes as MMT continues, such that eventually they no longer feel any significant progress physically.

Further, in our study the improvements in quality of life in the four domains were not always significant in the two post-baseline periods. Significant improvement in the environmental domain appeared at 6 but not 12 months, which is better than in mental health, where the scores were nonsignificant for the same groups of patients at both 6 and 12 months. Another possibility is that the effectiveness of MMT in improving heroin users’ quality of life may not be as great in Taiwan as has been found in other countries. Future studies should be carried out comparing countries at different time periods to examine the long-term and cross-cultural effects of MMT on heroin users.

In previous studies, perception of suitable costs and satisfaction of MMT are predictors of retention [12,25]. In other words, monetary costs and perception of improvement in QoL may both be critical factors in determining how long a patient stays in an MMT program. In our study, a patient in Taiwan has to pay $1,179.70 per year to participate in MMT based on regulations of Taiwan Department of Health. This cost from a patient’s perspective is surprisingly high, which result in the high cost per QoL increment in four domains.

In addition to the benefits in personal well-being uncovered in our study and others, MMT might also provide additional benefits. First, improvements in the four domains might increase the patient’s chances of obtaining or retaining a job. Second, family problems such as domestic violence or child abuse should be less likely to occur following the improvement in the patient’s quality of life. Finally, once the patient becomes less drug-dependent, the likelihood of involvement in drug-related crimes should be lower, a benefit to the society as a whole. Given that the direct and indirect economic costs associated with drug abuse are tremendous (more than $193 billion in the U.S. in 2007; see U.S. Department of Justice, 2011), and that MMT has been shown to be effective in reducing patients’ drug dependence, it follows that MMT should be considered a viable way to control and prevent the adverse consequences associated with drug abuse. Further, the costs of MMT are small compared with the social costs of criminal activity, loss of productive employment, and subsequent health care utilization on the part of active drug users. To reduce these drug-related economic losses, public policy makers should consider either completely subsidizing MMT or regulating it as a treatment for compulsive drug users.

This study employed a single-group prospective design which has methodological limitations in terms of causal inferences. First, we did not compare heroin users with a control group not in the MMT. The efficacy of MMT observed in this study may need to take threats of internal validity into consideration and, namely, the positive outcomes may be caused by other factors such as maturation, testing, and sample selection bias. Second, participant attrition has been shown to limit the generalizability of a study’s findings. Despite the large attrition rate in our study, no significant differences in demographics or clinical characteristics were found between individuals who completed at least 6 months of the study and those who dropped out before that. This outcome suggests that our findings are still valid. Third, the data were gathered through patient self-reports, where social desirability may be an issue. Future study may adopt alternative assessment strategies to maximize the disclosure of sensitive information; for example, ACASI – audio computer-assisted self-interview may be used to collect sensitive information. However, it is becoming increasingly important in the treatment of chronic diseases to use subjective quality of life measures as an outcome index.

Another limitation of our study is that the MMT costs listed in the Table 1 do not include all the direct and indirect costs. Note that this omission does not mean that every heroin user received all the treatments or examinations. In fact, whether such clinical examinations are performed depends on the psychiatrist’s professional decision regarding the appropriate methadone dosage. Moreover, we did not calculate opportunity costs such as the productivity loss from participating in an MMT program and the cost of police time spent dealing with crimes. We assumed that taking methadone everyday might have effects on a heroin user’s job performance, but the effects turned out to be rather negligible. Also, the costs for the time spent by police dealing with crime were difficult to estimate. Nevertheless, future studies are warranted to calculate the costs that are both directly and indirectly associated with MMT.

The results of the present study have important clinical implications for treating and retaining patients in MMT, particularly with respect to quality of life. Current clinical practice of MMT rarely implements the treatment and assessment of life of quality. Thus, we propose: 1) in addition to reduction in opioid drug use and HIV risk behavior, improvement in long-term quality of life can be of importance as an indicator of treatment outcome measure; 2) to carefully re-evaluate methadone maintenance dose prescribed in Taiwan. Previous study showed that higher methadone dose and longer duration of treatment were associated with better quality of life in MMT patients [29]. Higher methadone dose may reduce the severity of craving and relapse and retain in MMT longer [13]. Our findings showed that the average methadone dose prescribed in this Taiwan sample is lower than the recommended level. The re-evaluation and to prescribe appropriate methadone dose in Taiwan may be necessary in order to gain better quality of life for MMT patients; 3) to treat the comorbidity of other psychiatric disorders and infectious diseases, i.e. depression, anxiety disorder, and HIV/HCV is important to improve quality of life. Psychiatric comorbidity was as high as 78% in MMT patients [31]. Quality of life was worse in the patients of heroin dependence comorbidity of other psychiatric disorders [31] and among individuals with HIV [32]; 4) to establish a comprehensive psycho-social intervention in addition to medication to improve daily social function. Our results indicate the importance of longer duration in MMT with improvement of social relationship for patients. Compared to physiological and psychological well-being, increase in quality of social relations may need more time to show the effect. We suggest that social well-being can be one of the long-term clinical outcome measures.

## Competing interests

The authors declare that they have no competing interests.

## Authors’ contributions

YCC draft the manuscript and analyze the data; SFS and WDT contributed to the analysis, interpretation of the data and assisted in writing of manuscript; CRL contribute to the conceptual framework and interpretation of the results. KX assisted in interpretation of results. TSHL led research implementation and contribute to the conceptual framework, data acquisition, analysis and interpretation of the data for this manuscript. All authors read and approved the final manuscript.

## Pre-publication history

The pre-publication history for this paper can be accessed here:

http://www.biomedcentral.com/1471-244X/13/190/prepub

## References

[B1] National Institute on Drug Abuse Drug facts: Heroin: National Institute on Drug Abuse 2010 http://www.drugabuse.gov/publications/drugfacts/heroin

[B2] United Nations Office on Drugs and Crime World drug report 2012 2012 http://www.unodc.org/documents/data-and-analysis/WDR2012/WDR_2012_web_small.pdf

[B3] Bargagli AM Hickman M Davoli M Perucci CA Schifano P Buster M Brugal T Vicente J Drug-related mortality and its impact on adult mortality in eight European countries Eur J Publ Health 2006 16 198 202 10.1093/eurpub/cki168 16157612

[B4] Lejckova P Mravcik V Mortality of hospitalized drug users in the Czech Republic J Drug Issues 2007 37 103 118 10.1177/002204260703700105

[B5] Degenhardt L Hall W Warner-Smith M Using cohort studies to estimate mortality among injecting drug users that is not attributable to AIDS Sex Transm Infect 2006 82 Suppl 3 iii56 iii63 16735295 10.1136/sti.2005.019273 PMC2576734

[B6] Lee TS Shen HC Wu WH Huang CW Yen MY Wang BE Chuang P Shih CY Chou YC Liu YL Clinical characteristics and risk behavior as a function of HIV status among heroin users enrolled in methadone treatment in northern Taiwan Subst Abuse Treat Prev Pol 2011 6 6 10.1186/1747-597X-6-6 PMC3079677 21473789

[B7] Tan Y Wei QH Chen LJ Chan PC Lai WS He ML Kung HF Lee SS Molecular epidemiology of HCV monoinfection and HIV/HCV coinfection in injection drug users in Liuzhou, Southern China PLoS One 2008 3 e3608 10.1371/journal.pone.0003608 18974888 PMC2571986

[B8] Chen CY Wu PN Su LW Chou YJ Lin KM Three-year mortality and predictors after release: a longitudinal study of first-time drug offenders in Taiwan Addiction 2010 105 920 927 10.1111/j.1360-0443.2009.02894.x 20148787

[B9] Grella CE Lovinger K 30-year trajectories of heroin and other drug use among men and women sampled from methadone treatment in California Drug Alcohol Depend 2011 118 251 258 10.1016/j.drugalcdep.2011.04.004 21549528 PMC3156933

[B10] National Institute on Drug Abuse Drug facts: Understanding drug abuse and addiction: National Institute on Drug Abuse 2011 http://www.drugabuse.gov/publications/drugfacts/understanding-drug-abuse-addiction

[B11] O'Toole TP Pollini RA Ford D Bigelow G Physical health as a motivator for substance abuse treatment among medically ill adults: is it enough to keep them in treatment? J Subst Abuse Treat 2006 31 143 150 10.1016/j.jsat.2006.03.014 16919741

[B12] Kelly SM O’Grady KE Mitchell SG Brown BS Schwartz RP Predictors of methadone treatment retention from a multi-site study: A survival analysis Drug Alcohol Depend 2011 117 170 175 10.1016/j.drugalcdep.2011.01.008 21310552 PMC3148301

[B13] Villafranca SW McKellar JD Trafton JA Humphreys K Predictors of retention in methadone programs: A signal detection analysis Drug Alcohol Depend 2006 83 218 224 10.1016/j.drugalcdep.2005.11.020 16384657

[B14] Joseph H Stancliff S Langrod J Methadone maintenance treatment (MMT): a review of historical and clinical issues Mt Sinai J Med 2000 67 347 364 11064485

[B15] National Institute on Drug Abuse Drug facts: Treatment approaches for drug addiction 2009 http://www.drugabuse.gov/publications/drugfacts/treatment-approaches-drug-addiction

[B16] O'Connor PG Carroll KM Shi JM Schottenfeld RS Kosten TR Rounsaville BJ Three methods of opioid detoxification in a primary care setting: A randomized trial Ann Intern Med 1997 127 526 530 10.7326/0003-4819-127-7-199710010-00004 9313020

[B17] Gruber SA Tzilos GK Silveri MM Pollack M Renshaw PF Kaufman MJ Yurgelun-Todd DA Methadone maintenance improves cognitive performance after two months of treatment Exp Clin Psychopharmacol 2006 14 157 164 16756419 10.1037/1064-1297.14.2.157

[B18] Xiao L Wu Z Luo W Wei X Quality of life of outpatients in methadone maintenance treatment clinics J Acquir Immune Defic Syndr 2010 53 Suppl 1 S116 S120 20104102 10.1097/QAI.0b013e3181c7dfb5 PMC2818825

[B19] Centers for Disease Control Taiwan Monthly report on drug substance treatment (In Mandarin) 2012 http://www2.cdc.gov.tw/ct.asp?xItem=22061&ctNode=1885&mp=1

[B20] Laudet AB Becker JB White WL Don't wanna go through that madness no more: quality of life satisfaction as predictor of sustained remission from illicit drug misuse Subst Use Misuse 2009 44 227 252 10.1080/10826080802714462 19142823 PMC2629650

[B21] Maremmani I Pani PP Pacini M Perugi G Substance use and quality of life over 12 months among buprenorphine maintenance-treated and methadone maintenance-treated heroin-addicted patients J Subst Abuse Treat 2007 33 91 98 10.1016/j.jsat.2006.11.009 17588494

[B22] Padaiga Z Subata E Vanagas G Outpatient methadone maintenance treatment program. Quality of life and health of opioid-dependent persons in Lithuania Med (Kaunas) 2007 43 235 241 17413253

[B23] Ponizovsky AM Grinshpoon A Quality of life among heroin users on buprenorphine versus methadone maintenance Am J Drug Alcohol Abuse 2007 33 631 642 10.1080/00952990701523698 17891656

[B24] De Maeyer J Vanderplasschen W Broekaert E Quality of life among opiate-dependent individuals: A review of the literature Int J Drug Pol 2010 21 364 380 10.1016/j.drugpo.2010.01.010 20172706

[B25] Yang F Lin P Li Y He Q Long Q Fu X Luo Y Predictors of retention in community-based methadone maintenance treatment program in Pearl River Delta, China Harm Reduct J 2013 10 3 10.1186/1477-7517-10-3 23497263 PMC3599968

[B26] The WHOQOL Group Development of the World Health Organization WHOQOL-BREF quality of life assessment Psychol Med 1998 28 551 558 9626712 10.1017/s0033291798006667

[B27] WHOQOL-Tawian Group Introduction to the development of the WHOQOL—Taiwan version (In Mandarin) Chin J Publ Health 2000 19 315 324

[B28] Baharom N Hassan MR Ali N Shah SA Improvement of quality of life following 6 months of methadone maintenance therapy in Malaysia Subst Abuse Treat Prev Pol 2012 7 32 10.1186/1747-597X-7-32 PMC3570287 22853701

[B29] Wang PW Wu HC Yen CN Yeh YC Chung KS Chang HC Yen CF Change in quality of life and its predictors in heroin users receiving methadone maintenance treatment in Taiwan: an 18-month follow-up study Am J Drug Alcohol Abuse 2012 38 213 219 10.3109/00952990.2011.649222 22352836

[B30] Ha NTT The effect of methadone maintenance treatment in improvement of quality of life for heroin users in Hai Phong, Vietnam The 4th International Conference on Reproductive Health and Social Sciences Research. Bangkok, Thailand 2010

[B31] Carpentier PJ Krabbe PF van Gogh MT Knapen LJ Buitelaar JK de Jong CA Psychiatric comorbidity reduces quality of life in chronic methadone maintained patients Am J Addict 2009 18 470 480 10.3109/10550490903205652 19874168

[B32] Lee TSH Shiu CS Tuan YC Wu WH Huang CW Yen MY Lin CH Shen LC Lin CK Quality of life among injection drug users living with or without HIV/AIDS in Taiwan: A case control group design J AIDS Clin Res 2013 4 3

